# Easy Access to Antibiotics; Spread of Antimicrobial Resistance and Implementation of One Health Approach in India

**DOI:** 10.1007/s44197-021-00008-2

**Published:** 2021-09-28

**Authors:** Kunal Jani, Vibhaw Srivastava, Preeti Sharma, Aruna Vir, Avinash Sharma

**Affiliations:** DBT Wellcome Trust Fellow, DBT-National Centre for Cell Science, Pune-411021, India

**Keywords:** Antimicrobial resistance, Mass gatherings, AMR containment policy, One health approach

## Abstract

Antimicrobial resistance (AMR) is a global public health concern because of its fast spread. India, one of the world’s top consumer of antibiotics and second most populated country has its unique constraints of social, cultural and economic strata. The continual self-medication, use of antibiotics for the growth promotion in animals, and accumulation of residual antibiotics in the environment challenge the implementation of AMR containment policy. Hence, the present review attempts to delineate the influence of antibiotics abuse on the human, animal and environmental health under the realm of one health. It was based on the literature search using public databases to highlight the rapid surge in the burden of AMR in India affecting various sectors and/or ecosystems in India. It was found that the irrational and overuse of antibiotics in different sectors have led to the emergence of extended antimicrobial resistance wherein the environment acts as a reservoir of antibiotic resistance genes (ARGs); completing the cycle of contamination and recontamination. There are efforts by government policy makers to reduce the burden of AMR in the country to reduce the health risks, through the One Health approach. Parallel efforts in educating healthcare professionals, strict legislation for pharmacies and pharmaceutical companies should be prioritize. At the same time surveillance of newly emerged AMR pathogens, prioritising research focusing on AMR, and awareness camps or programs among the local population is critical while addressing the consequences of spared of AMR in India.

## Introduction

Discovery of the antimicrobial agents in the early twentieth century was a breakthrough to safeguard the public health from the microbial infection. Propensity of these compounds to kill pathogens influenced the discovery of novel antimicrobials. However, since the 1970s, soon after the discovery of the fluoroquinolones, no major antibiotics have been introduced [1]. Moreover, the accessibility and availability of antibiotics results in irrational and overuse of antibiotics contributing to antimicrobial resistance (AMR). Non-prudent use of antibiotics accompanied by genetic plasticity allows microorganisms to adapt to the effect of antimicrobials causing a rapid surge of antimicrobial resistance [2, 3]. The resultant increase in antimicrobial resistance obstruct action of antimicrobials which in turn risk the human health. Further, self-medication, over the counter availability of antibiotics, prescription of broad-spectrum antibiotics and negligence towards the dosage depict the responsible factor for the increase in the antibiotics exposure and growing AMR [2, 6].

Bacteria are ubiquitous in nature; thriving in soil, water and air and, interconnectedness of ecosystems, featuring human, animals and environment collectively indicates that the burden of AMR is multifaceted in nature [2]. Antibiotic selection pressure is fundamental in the development of resistance in bacteria. In addition, the phenomenon of horizontal gene transfer (HGT), transformation, transduction or conjugation carry genetic elements, elevating antimicrobial resistance and virulence in microorganism [7–9]. Moreover, as the antimicrobial resistance is transmissible among the microorganisms, therefore bacterial strains acquire more than one antibiotic resistance gene (ARG). The perpetual exchange and acquisition lead to the generation of a pool of ARGs in the environment, result of which is responsible to turn a non-pathogenic bacteria into a multidrug-resistant strain and thereby outbreak of infectious disease occurs. Majority of the antibiotics work by impairing bacterial protein synthesis, interestingly the stress of antibiotics also dramatically induces the toxin-antitoxin system mediated generation of ‘persister cells’ [4, 5]. These persister cells represent the subpopulation of community which could resist the impact of antimicrobials and subsequently induce reoccurrence of disease. Thus, antibiotics abuse along with paucity in technological progress is threatening global health, as it struggles to cope-up with the evolutionary rate of pathogenic bacteria.

**Fig. 1:**
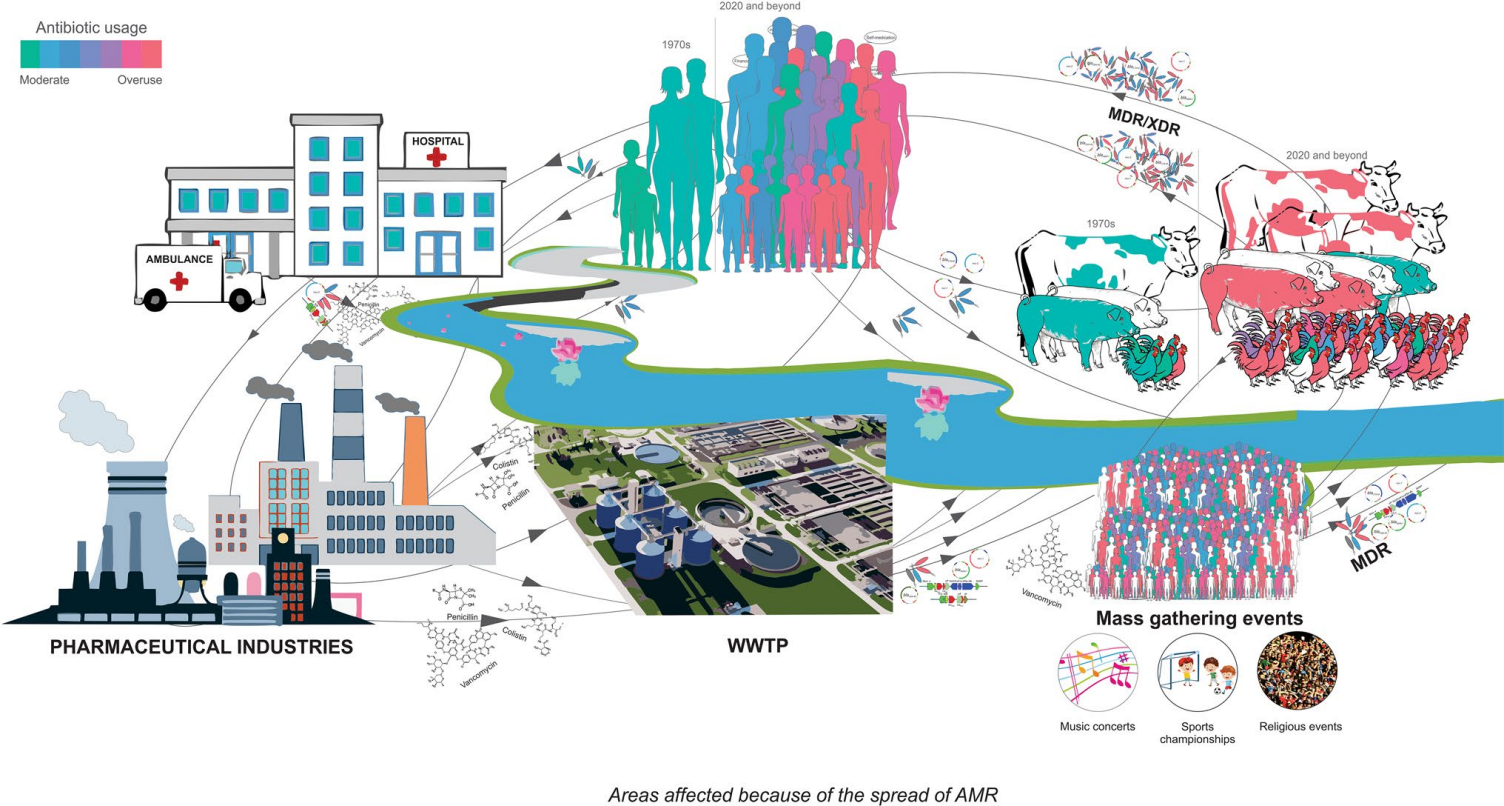
Spread of AMR in various sectors of India

The World Health Organization (WHO) categorize antibiotics as essential medicines and suggest their availability at affordable prices to meet the priority healthcare needs. Additionally, WHO has also developed a scheme to monitor the usage of antibiotics called AWaRe i.e. Access, Watch, Reserve (AWaRe) classification of antibiotics. Under this program, the antibiotics usage is timely evaluated and monitored to reduce the rampant spread of AMR pathogens [10]. Whilst, a report by Klein, indicates an unusually high rate of consumption of antibiotics by low and middle-income countries (LMICs) than high-income countries [9]. A systematic follow-up in LMICs during 2000–2015 revealed 114% rise in antibiotic consumption and 77% increase in the rate of antibiotic consumption [9]. Specifically, the BRICS countries i.e. Brazil, Russia, India, China and South Africa collectively contributed for a 76% rise in the global antibiotic consumption during the year 2000–2010 [11]. Unfortunately, India is among the highest consumers of antibiotics with as high as 23% increased retail sales when compared to other BRICS countries [11]. Additionally, with the discovery of *bla*_*NDM-1*_ (New Delhi metallo-β-lactamase) and associated controversy necessitated the policymakers to initiate the development of AMR containment-related policies for India in 2011 [12–14]. Specifically talking about India, excessive usage of antimicrobials is not restricted to the humans, but also common in food animals driven by the need for higher milk and meat production and aquacultures [15–20]. Therefore, it has been recommended to address the AMR issue with the broader perspective of one health approach. As the Centers for Disease Control and Prevention (CDC) defines “One Health is a collaborative, multisectoral, and transdisciplinary approach- working at the local, regional, national, and global levels with the goal of achieving optimal health outcomes recognizing the interconnection between people, animals, plants, and their shared environment” (https://www.cdc.gov/onehealth/index.html) (Fig. 1). Thus, considering the interconnected nature of the animals, humans, and environment and, the level of complexity involved in the interactions; would demand multi-centric, collaborative and global efforts for the effective containment of AMR and associated anomalies.

Antibiotic selection pressure is known to be a key component modulating the rise in AMR however, in the Indian scenario it would be imperative to take into account the influence of social (including economical strata), cultural and ecological factors. Hence the current review provides an overview of the growing AMR burden in humans, food, animals and environment from one a one health approach.

## Methodology

The current review was developed from the available literature related to the spread of antimicrobial resistance in India. The intensive literature search was carried out by using public databases viz. Google scholar and PubMed. The search was made using the combination of keywords like, “antimicrobial resistance”, “antibiotic resistance genes”, “fisheries”, “antimicrobial resistance in animals”, “antibiotic abuse in India”, “one health”, “antimicrobial resistance mass gathering events” and “antibiotic resistance genes in the environment”. The search was narrowed to articles published between 2010 to 2020. The articles providing details on each of these topics (or in combination) were filtered and used for the synthesis of this review. The review was aimed to provide the overview of the growing issue of AMR in India from the one health approach.

## Major Areas Affected by Overuse of Antibiotics

### Human

Globally, India has the highest burden of infectious diseases [3, 21]. In particular, as high as 50% of children aged ≤ 5 years die as a result of pneumonic and diarrheal infections [21]. Additionally, the rate of infectious diseases also correspond to the higher volume of sales and antibiotic consumption in the country [21]. Factors affecting antibiotic consumption are either institutional (hospital or pharmaceutical companies) or individual (doctors and/or patients). They include lack of diagnostic facilities, irrational prescription of antibiotics, lack of knowledge and experience about appropriate use of antibiotics and incentives from pharmaceutical companies for the profitable drug sales together accounts for the dereliction of duties by healthcare institutions [21, 22]. The financial burden of healthcare instigates patients to avoid the diagnostic tests, over the counter purchase of antibiotics i.e. indulging in self-medication by using older prescription or leftover medicines, encourages impatient behaviour (to have a rapid recovery) and negligence toward the prescribed dosage, collectively contributes for irrational use of antibiotics and eventually increased AMR [22, 23]. Moreover, a study published in the Lancet raises concern about the effectiveness and safety of the drugs used for the treatment [24]. It details the challenges faced by Central Drugs Standard Control Organisation (CDSCO), a drug approving and licensing body, including understaffing and overburdened. Because of this, only 42 drugs were approved in 2012, 33 of which were not supported by any scientific or safety data. In fact, 11 out of 42 were lacking phase 3 trials. Additionally, 13 drugs were approved for its sale in India, despite of its banned across other developed nations [24]. Unfortunately, individual healthcare providers especially those in rural settings are often incompetent and employ staff who lack formal training. Hence, private healthcare is preferred burdening individuals for out of pocket payments, especially to the low-income earners [25]. Thus, to minimize the healthcare expenditure, people indulge in self-medication leading to increase in per capita consumption of antibiotics. The observation corroborates the findings of Wu et al. (2021), depicted the increase in perceived barriers related to increased self-medication by parents to their children (age ≤ 5 years), which represents a daunting challenge to ensure the health of the children [26]. Subsequently, it promotes the antimicrobial resistance which corroborates the observed higher resistance towards the first generation to broad-spectrum antibiotics [2].

**Fig. 2:**
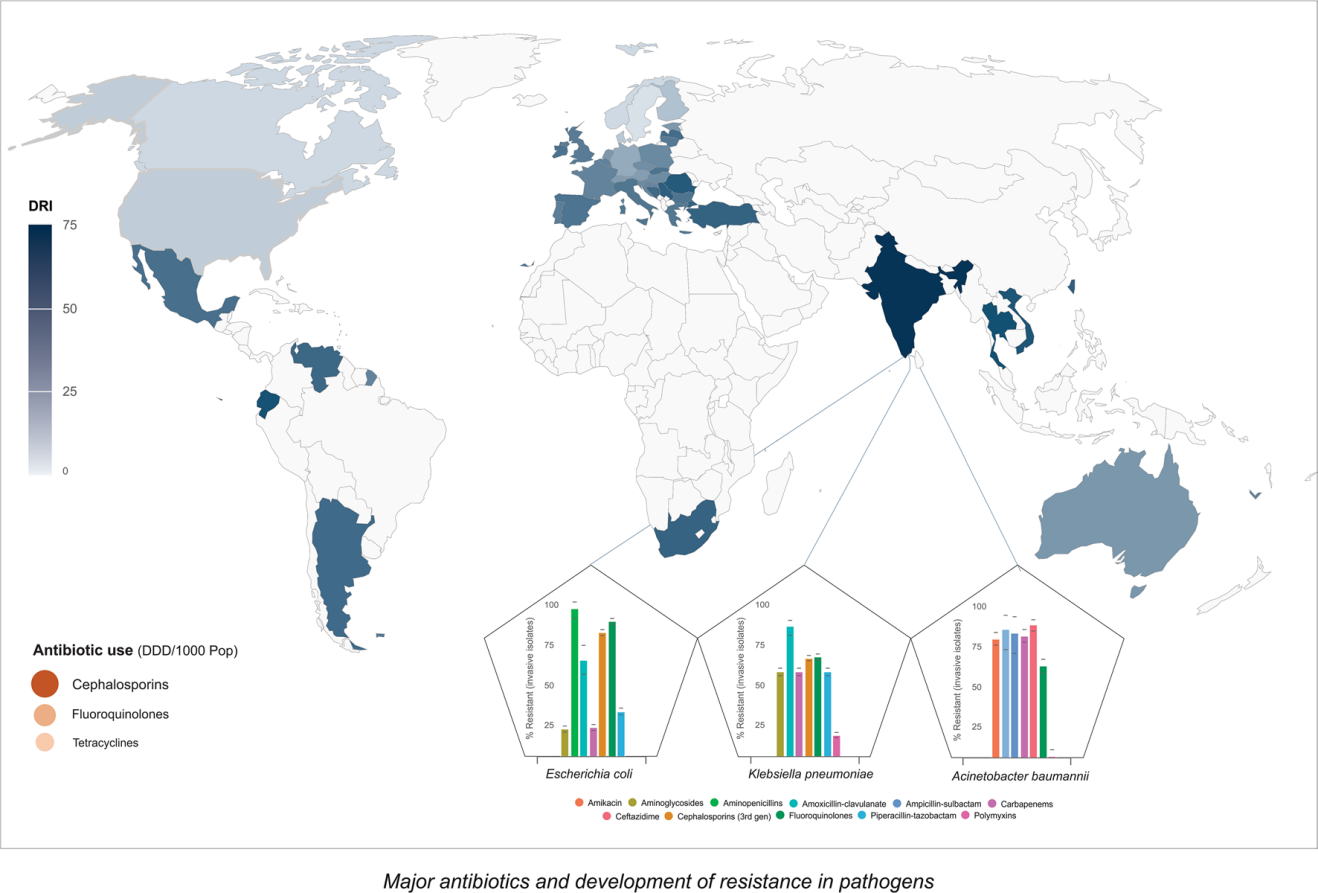
Major antibiotics and development of resistance in pathogens

*Acinetobacter baumannii, Escherichia coli, Klebsiella pneumoniae* and *Pseudomonas aeruginosa* have been found to confer higher resistance to fluoroquinolones and third-generation cephalosporin and colistin. Ineffectiveness of last resort antibiotic pose a serious concern to safeguard the public health (Fig. 2) [25]. Moreover, the emergence of gram-positive bacteria such as methicillin-resistant *Staphylococcus aureus* (MRSA) and *Streptococcus pneumoniae* present additional challenges. Interestingly, in the case of *Salmonella Typhi,* resistance to ampicillin and trimethoprim-sulfamethoxazole was found to decrease by ~ 2.4 and ~ fourfold, respectively [27]. Thus, re-introduction of first-generation antibiotics over extensively prescribed broad-spectrum antibiotics could help to evade such enteric pathogens. India has become a hub to multidrug-resistant (MDR) and extensively drug-resistant (XDR) *Mycobacterium tuberculosis* which also demands efforts to mitigate onset of tuberculosis. Additionally, the genomic plasticity of these organisms enabling them to develop resistance against the last-resort antibiotics like Carbapenemases and Colistin by recombination or genetic acquisition may represent a daunting task, as the available antibiotics would no longer be effective against these organisms. The New Delhi metallo-beta-lactamase-1 (*bla*_*NDM-1*_) depicts one such genetic element providing resistance against Carbapenemases. Prevalence of such antibiotic resistance genes (*bla*_*NDM-1,*_* bla*_*OXA-48*_*. mcr-1 and mcr-2*) in the environment and their possible transmission renders an alarming need to formulate action plans to ensure public health [28, 29].

### Animal Husbandry Including Aquaculture

The ever-increasing demand of animal-based food products compelled the producers to increase the usage of antibiotics and growth-promoting factors in the animal feed. The higher usage of antibiotics aids the better health of animals and also enable industries to abandon the responsibilities of maintaining hygiene and sanitation. Such ill-usage of antibiotics in the animal feed leads to bioaccumulation which was supported by observed traces of antibiotics in animal-based food products [3]. Majority of studies on the poultry farms depicts the presence of various antimicrobial resistant organisms conferring resistance to one or more antibiotics [15, 31–33]. Taxonomic identification of these poultry isolates represents a higher frequency of isolates affiliated to *E. coli* or *Salmonella* species. Furthermore, there have been molecular investigations suggesting extended-spectrum beta-lactamase (ESBL)-producing and colistin-resistant *E. coli* from the poultry [33]. Moreover, the Antibiotics that are used for the treatment of humans are commonly used in animals, and this is important for public health prospective [2]. The antibiotic-resistant bacteria can be transmitted to the humans through direct contact with animals and through consumption of contaminated foods which further elevates the risk to public health [30]. Moreover, bioaccumulation of residual antibiotics in animal-based food products further threaten the public health by elevating the risk of transmission of ARGs among the resilient gut-microbiota.

MDR strains like Vancomycin-resistant *Staphylococcus aureus* have been isolated from the animals suffering from mastitis [34]. Further, cow, goats and buffaloes have been found to carry strains resistant to ampicillin, ceftriaxone and enrofloxacin, oxacillin and vancomycin [35, 36]. Moreover, it was devastating to note the persistence of gram-negative ESBL producing microbes from the cattle meat and milk [25]. Similarly, the strains isolated from the raw milk and the fecal samples of pig were resistant against ampicillin, streptomycin, oxytetracycline and to some extent to chloramphenicol and trimethoprim sulfamethoxazole [35]. Excessive antimicrobial use has also been reported in aquaculture [2, 17]. Studies focusing on the coastal part of India demonstrated various gastrointestinal infections due to *Vibrio* species and members of family *Enterobacteriaceae* [16, 17]. Prevalence of such pathogenic microorganisms was not restricted to the water bodies (lakes or sea) whilst studies have shown their prevalence in a variety of aquatic biota including fishes, shrimp and shellfish [16]. Additionally, these microbial strains depict similar traits of resistance towards ampicillin, trimethoprim sulfamethoxazole and vancomycin, as noted in the animals [15–17, 33].

### Environment and Human Mass Gatherings

The finding of antimicrobial-resistant microorganisms in humans, poultry, cattle and fishes reveal possible transmission and sharing of such microorganisms and antibiotic resistance genes (ARGs) between these organisms. However, the underlying mechanism of transmission and establishment of AMR is not well understood [37]. Thus, a higher degree of environmental contamination, due to discharge of antibiotics, leads proportionately higher frequency spread of AMR. Water bodies including lakes and rivers receive loads of untreated sewage and industrial wastes, dramatically reducing the quality of drinking water [38].e.g. Germany had suffered the catastrophic outbreak of gastrointestinal infections during the year 2011 due to contamination of drinking water with multidrug-resistant microorganisms [39].

Sewage plants receiving untreated or inadequately treated hospital and industrial effluents are a potential reservoir of AMR and ARGs. Discharge of untreated sewage into the rivers is a another major factor contaminating the river and facilitating the spread of AMR. Recent studies from India have indicated that the hospital waste carries significantly higher proportion of antibiotic residues, particularly the traces of broad-spectrum antibiotics [40]. In the hospital intensive care units (ICUs) broad-spectrum antibiotics such as third-generation cephalosporins or carbapenemases are frequently prescribed to minimise nosocomial infections. There have been various single time point studies in India employing culture-dependent approaches to infer the potential of microbes in the growing AMR [21, 52]. Additionally, molecular investigation of potential AMR strains suggested presence of extended-spectrum beta-lactamase (ESBL) and extended-spectrum cephalosporins (ESC) producing organisms. The AMR potential was reconfirmed by detecting the presence of *bla*_*NDM-1*_*, bla*_*OXA-48*_*, bla*_*CTX-M*_ and *mcr-1* genes [41–43]. bla_*NDM-1*_ genes are responsible for the development of drug resistance in pathogens and also provide an advantage to evolve as a superbug [44]. Similarly, the lack of strict regulatory measures in place for pharmaceutical industries has led to the release of tons of ineffectively treated effluents into the river or nearby water bodies [19, 41]. The key caveat is that countries have developed good manufacturing practices for pharmaceutical companies by joining hands with WHO, whilst it lacks the effective implementation of the regulations. There are two main categories of pharmaceutical companies including (1) active pharmaceutical ingredient manufacturer (API) responsible for bulk production of antibiotics and (2) formulation companies who technically depend on API to manufacture final products. The industrial effluents from both categories of pharmaceutical companies significantly contaminate the water bodies. The Central Pollution Control Board (CPCB) is said to lack policies to monitor and control the discharge of residual antibiotics in the effluents [2].

India is a country of festivals involving several cultural events during the year, one such event is the Kumbh Mela. It is considered as the world’s largest mass gathering event attracting millions of pilgrims for the duration of three months [45, 46]. The event involves a ritual bathing in the river, wherein bathing by the millions of attendees may have serious consequences on the river as well as on public health. The is known to involve participation by people from varied socio-economic backgrounds who may practice varied levels of hygiene practices, providing a good ground for the exchange of AMR and ARGs. Moreover, lack of facilities to accommodate such a huge number of pilgrims with inadequate supply of clean water and sanitation results in the spread of infectious diseases [47]. The Kumbh Mela have been known to be associated with several outbreaks of gastrointestinal diseases and skin infections and, subsequently sporadic [45, 47]. Waste disposal is another concern and open-defecation by those in attendance has been reported. The leaching from untreated solid and sewer waste into the river body may lead to a surge in faecal coliform affecting the water portability. In a recent study, the persistence and abundance of *bla*_*NDM-1*_ were monitored during the seasonal pilgrimage on the bank of Ganga. The authors reported a 20-fold increase in prevalence during the pilgrimage [48]. Similarly, the study focusing on the 2015 Kumbh Mela event held in Nashik, India revealed the presence of substantially higher abundance of gene families conferring drug resistance and infectious diseases [49, 50]. Antibiograms of bacterial strains belonging to genera *Acinetobacter, Corynebacterium* and *Brevibacterium* which was isolated during the event show higher antimicrobial resistance [20]. *Corynebacterium godavarianum,* a novel bacterium isolated during the Kumbh Mela 2015, was found to confer resistance against an array of antibiotics supporting the observed increase in antimicrobial resistance during the mass bathing event [51].

India is one of the ~ 184 countries that take part in Hajj pilgrimage at the Mecca, Saudi Arabia. Participation of pilgrims from these diverse countries has been associated with the morbidity and mortality at the event. In past, the Hajj has witnessed the burden of communicable diseases like, *Neisseria meningitidis* serogroup W135 in 1968, 1987, 2000 and 2001, H1N1 pandemic in 2009, Middle East Respiratory Syndrome Coronavirus (MERS-CoV) in 2012, etc. Additionally, such a large participation also elevates the risk of stampedes or crush injuries, hence necessitates intensive planning and preparedness of the event to minimize the risk to the public health [50–54]. Mass gatherings due to social and sports events also strain the host nation, in planning and executing these activities, and can be quite demanding. [50, 52, 55]. The participants are from diverse socio-economic backgrounds with varied degrees of hygiene which could play a role in the transmission of antibiotic-resistant microorganisms [50, 52]. Thus, it is critical to have a detailed record of people attending these mass gatherings, monitoring their movement and also continuous surveillance to minimise the events of contamination and thereby lowering the risk of infection.

### One Health

The striking observations of threatened humans, animal husbandry and fishery and, environment health due to growing antimicrobial resistance raise a serious concern (Fig. 1). Self-medication, incomplete dosage and preference towards the third generation and/or combination of antibiotics were found to risk the human health [21–23]. Subsequently, the discharge of antibiotic residues from the households in the form of sewer waste and effluents from the pharmaceutical industries elevates the levels of antibiotics in the environment [38–42]. Contamination of the water bodies leads to the accumulation of antibiotics in the fish another example of biomagnification. Similarly, human antibiotics are administered in poultry and animal breeding to minimize the cost of sanitation increase the exposure of antibiotics [31–33]. Consumption of such contaminated meat and meat products could lead to the spread of ARGs. The complex nature of the interaction between the humans, animals, microorganisms and their environment necessitates strategic planning and implementation using one health approach to combat the spread of AMR [3]. As the one health approach prioritize the interlink between different sectors over their individuality, it is capable of providing insight into the mechanism of transmission of antimicrobial resistance between the groups. Moreover, such global approach would help derive the relative contribution of different segments in the growing reservoir of ARGs. Alternatively, it is also crucial to take into account the external stimuli and evolutionary measures empowering the genetic variability among the microbial populations [6–8, 56]. Studies have also shown that the antibiotic abuse by humans and/or for the production of farm animals exceeds the rate of evolutionary genetic modifications [56]. Later, enforces coding of an intrinsic ARGs by the persister cells in a given microbial community or acquired ARGs from the available gene pool. Given the interconnectedness, unfortunately, there’s no control over the exchange of ARGs between the organismal and environmental system. Additionally, due to the malpractices of antibiotic usage and its bioaccumulation in human, animals and environment there is logarithmic expansion of ARG gene pool [57]. Despite the availability of cutting-edge technology and awareness among the researchers about this alarming issue of AMR, the containment of AMR spread represents a major challenge. In contrast, the studies are reporting newer variant of MDR and XDR strains with the presence of integron gene cassettes or plasmids [43]. Additionally, deriving conclusions from the controlled environment studies and its implementation to an environment, experiencing continuous shift in the chemistry as well as microbial population seems far-fetched [43, 44]. Thus, its crucial to consider all the segment of organismal and environmental system at once to contain and mitigate the issue of AMR spread in India.

## Initiatives of Indian Government in Tackling the Overuse of Antibiotics

To address the ever-growing challenge of antimicrobial resistance and to safeguard public health, officials keep on upgrading the local policies by following the strategies outlined by WHO. The controversies related to the NDM-1 drew the attention of policy makers to initiate the National Policy on Containment of AMR in 2011 [58]. The Ministry of Health and Family Welfare (MoHFW) established a National Task Force on AMR Containment in 2010 with the primary focus of not only assessing the AMR situation in the country but also draw a roadmap for the country (Table 1). Subsequently, there were ample amendments in planning and management of AMR containment by following the Jaipur Declaration (2011) and Chennai Declaration (2012) [59–61]. The government gradually increased the stringency to minimize over-the-counter sales of antibiotics as well as restricting the use of third-generation and broad-spectrum antibiotics. In 2012, a five-year national plan was developed under the governance of National Centre for Disease Control (NCDC) with primary focus on the establishment of nationwide AMR surveillance. This initiated interaction between healthcare professionals, laboratories, individuals, policy makers and other key stakeholders. Parallelly, Antimicrobial Stewardship, Prevention of Infection and Control (ASPIC) was launch to educate and increase awareness on the rational use of antibiotics [62]. In the year 2014, there was an implementation of Schedule H1 to regulate the purchase of antibiotics. Recently, an Indo-US commitment i.e. Global Health Security Agenda (GHSA) was established to monitor and mitigate the issues of AMR.

**Table 1:** Key events in AMR policy making in India

Year	Policy implications	Objective
2010	Established a National Task Force on AMR Containment	Assessment of the AMR situation in India
2011	Jaipur Declaration	Amendments in planning and management of AMR containment
2012	Chennai Declaration	
2011	The Food Safety and Standards (Contaminants, Toxins and Residues) Regulations, by FSSAI	Regulation on usage of antibiotics in food animals
2012	National Centre for Disease Control (NCDC)	5-year national plan for the AMR surveillance
2014	Implementation of Schedule H1	Minimize the over the counter availability of the certain antibiotics
2016	Launch of the Red Line Campaign on Antibiotics to create awareness regarding rational usage of antibiotics	Awareness program to educated the people for rational usage of antibiotics
2017	Delhi Declaration	Amendments in planning and management of AMR containment
2017	The Food Safety and Standards (Contaminants, Toxins and Residues) Regulations in food animals	

The table content was adapted and modified from the Gandra et al. [2]

Similarly, to safeguard the animal health, Food Safety and Standards Authority of India (FSSAI) has developed norms for the rational use of antibiotics. The FSSAI has banned the use of several antibiotics that could harm the fisheries and those used in the seafood processing [63]. Furthermore, it included guidelines for the for the usage of permissible antibiotics in seafood processing and for the production of honey. However, there are no specific regulations for the use of antibiotics in poultry, animal rearing and environmental health. The latest, National Action Plan for Containment of AMR (NAP-AMR) aims to improve the awareness about AMR, strengthening surveillance in various sectors, regulation for optimized use of antibiotics, promote research in this area, and establish new international collaborations [61]. Although, there have been great efforts to devise the strategies to mitigate the growing issue of AMR, however, its effectiveness is undefined. Especially, it lacked measures to monitor and examine the effectiveness of the AMR containment policies. For instance, despite to regulation of Schedule H1 in place antibiotics were still available without medical prescription [64]. Likewise, implications of antibiotics awareness under the Red Line Campaign remains unclear. Finally, the composite and perpetual efforts by the Government of India to combat the AMR spread are commendable. As it will eventually help to contain the spread of AMR in India.

## Conclusion

The irrational and overuse of antibiotics in different sectors of India i.e., humans, food, animals and environment are responsible for the ever-increasing antimicrobial resistance (AMR), however, it has received little attention compared to its drastic impacts. Mitigation of the growing antimicrobial resistance would require strategies focusing on the one health approach. Initiative of the Indian government such as NAP-AMR policy along with the continued surveillance, awareness camps, and cross-continental research and training would enable combating the issue of AMR. Initiatives like designated AMR repositories by the Department of Biotechnology, Government of India at the National Centre for Cell Science, India would be helpful to generate and validate the emergence of AMR-related data in different sectors of India.

## Data Availability

Not applicable.
